# Effect of one or two fixed glutamate doses on follicular development, ovarian-intraovarian blood flow, ovulatory rate, and *corpus luteum* quality in goats with a low body condition score

**DOI:** 10.1590/1984-3143-AR2022-0117

**Published:** 2023-12-11

**Authors:** Alfredo José Herrera Conde, Juliana Paula Martins Alves, César Carneiro Linhares Fernandes, Maria Raquel Lopes Silva, Camila Muniz Cavalcanti, Alessandra Façanha Bezerra, Dárcio Ítalo Alves Teixeira, Davide Rondina

**Affiliations:** 1 Faculdade de Veterinária, Universidade Estadual do Ceará, Fortaleza, CE, Brasil; 2 Faculdade de Medicina Veterinária, Universidade de Fortaleza, Fortaleza, CE, Brasil

**Keywords:** goat, glutamate, ovary, corpus luteum, Doppler

## Abstract

This study aimed to investigate the effect of a short glutamate supply on the ovarian response in goats with low body condition scores. Twenty-one goats had their estrus and follicular waves synchronized using three injections of prostaglandin analog at seven-day intervals. Goats were allocated to groups receiving 10 mg/kg LW (iv) of glutamate administered in a single dose (group LBCG1, n = 7) or in two doses five days apart (group LBCG2, n = 7). The control group (LBC; n = 7) received saline solution. Glutamate treatment did not affect glucose, cholesterol, or glutathione peroxidase levels, body weight, or adipose deposits. During the experimental period, the LBCG2 group showed a higher (P < 0.05) number of follicles (> 3 mm) and an increase in follicle diameter (P < 0.05). Glutamate supply improved (P < 0.05) the intraovarian Doppler blood area size in the LBCG groups, and the second dose in LBCG2 also induced a higher (P < 0.05) systolic and diastolic peak of the ovary artery. After ovulation induction, LBCG2 exhibited a high (P < 0.05) volume of the corpus luteum and vascularized area. We concluded that the supply of two doses of glutamate five days apart was efficient in ovarian stimulation in goats with a low body condition.

## Introduction

Research on mammals using specific nutrients, including amino acids (AAs), has gained momentum in the last decade, opening new routes for studying the relationship between nutrition and animal reproductive response (Maffucci and Gore, 2009; Sugimoto et al., 2010). In ruminants, AAs such as glutamate are one of the main signals of stimulation, the pulsatility of GnRH, the control of the LH peak, and consequently ovulation induction (Dhandapani and Brann, 2000). Thus, the theme is relevant because the metabolic state is a powerful signal at several crucial moments of the animal reproductive life, such as puberty or the resumption of cyclic activity in the postpartum period (D’Occhio et al., 2019).

In non-ruminant species, AA supplementation can occur through diet. In ruminants, in the other hand, alternative strategies should be adopted because microbial metabolism is variable with regard to the remodeling of ingested nutrients for amino acid synthesis (Gilbreath et al., 2021). In goats, l-glutamate supplementation has been performed through intravenous administration at various dosages and periods, depending on the intended reproductive objective (Torres-Moreno et al., 2009; Meza-Herrera et al., 2014b, 2020).

In goats, glutamate can act on centers that promote reproductive activity during puberty, the estrous cycle, and seasonal anestrus. Two doses of l-glutamate supplementation were tested in goats: 0.175 mg/kg LW of l-glutamate, which improved the ovulation rate in cyclic goats (Meza-Herrera et al., 2014b), and 7 mg/kg LW of l-glutamate, which stimulated ovarian activity, induced an early onset of puberty (Torres-Moreno et al., 2009; Meza-Herrera et al., 2011), decreased the negative feedback effects of estradiol (E2) (Meza-Herrera et al., 2020), and increased LH pulses (Luna-García et al., 2022). In addition, the aforementioned dose improved reproductive behavior and male effects in rams (Calderón-Leyva et al., 2018) and positively affected their sperm production during the natural period of sexual rest (Calderon-Leyva et al., 2017).

Studies have reported that glutamate administration does not interact with metabolites such as insulin (Torres-Moreno et al., 2009), glucose (Meza-Herrera et al., 2011), total protein, and urea nitrogen (Meza-Herrera et al., 2011, 2014a). However, the results are still contradictory, as other studies have reported an increase in insulin and triiodothyronine (Meza-Herrera et al., 2011, 2020), in addition to cholesterol (Meza-Herrera et al., 2014a). This low variation in blood metabolites is partly justified because these studies were conducted with animals under ideal nutritional conditions. As described by Viñoles et al. (2010), animals with an ideal body condition score (BCS) do not show variations in metabolite concentrations even when subjected to food supplementation. Therefore, verifying the effectiveness of glutamate administration under different nutritional states is necessary. In addition, the concentration of glutamate and the time of its administration in small ruminants remain uncertain.

Nutrition determines the metabolic state that promotes the activity of intraovarian factors (glucose-insulin system, leptin, and IGF1) that stimulate the function of the hypothalamic-pituitary-ovarian (HPO) axis (Scaramuzzi et al., 2006). The effects of nutrition on the ovulatory response can be clearly observed in animals with high BCS. The reason for this phenomenon is that ovarian activity improves when the concentrations of metabolic hormones (insulin and IGF1) increase and remain high, an effect not observed in animals with low BCS (Viñoles et al., 2010). Low BCS results in a low concentration of metabolic hormones (IGF1) (Viñoles et al., 2010), causing the low production of E2 by the follicles (Scaramuzzi et al., 2011). The negative feedback caused by low E2 concentrations limits glutamate activity in the hypothalamus (Porter et al., 2021), resulting in low hypothalamic activity for GnRH release (Rhind et al., 1989). However, in undernourished animals, the pituitary gland remains sensitive to GnRH and can produce gonadotropins for follicular growth and ovulation (Rhind et al., 1989; Meza-Herrera et al., 2007).

The hormonal effect directly affects the reproductive behavior of mammals, as it results from the interaction between the central nervous system and the reproductive system through the activity of reproductive hormones, receptors, and glutamate, which are the main excitatory neurotransmitters (Chiang and Park, 2020). Progesterone (P4) and E2 induce different neuroendocrine conditions in the brain; P4 inhibits the expression of glutamate receptors (AMPA, Kainate), while E2 promotes the expression of glutamate receptors (NMDA) (Barth et al., 2015). Porter et al. (2021) observed that during the follicular phase of sheep, an increase in E2 favors the transport of glutamate, which probably contributes to the positive feedback responsible for the secretion of GnRH and the pre-ovulatory LH surge.

To improve ovarian activity during the start of breeding season, glutamate supplementation can improve the reproductive performance of goats that still have low BCS. According to Gilbreath et al. (2021), the use of AAs can be a strategy that improves fertility and promotes reproductive efficiency in the ruminant production sector. In the literature, it has been reported that, administration of l-glutamate decrease the effects of negative feedback on hypothalamic centers that suppress GnRH release (Meza-Herrera et al., 2020; Luna-García et al., 2022).

Thus, we hypothesized that the intravenous application of glutamate would be effective in stimulating the ovarian response in animals with low nutritional status when administered in a single dose or two doses with an interval of five days.

Therefore, this study was designed to determine whether the supply of one or two glutamate doses administered five days apart influenced glucose, cholesterol concentration, glutathione peroxidase levels, ovarian-intraovarian blood flow, follicle development, ovulatory rate, and quality of corpus luteum in goats.

## Methods

### Location and animal ethics

This study was conducted at the facilities of the laboratory of nutrition and ruminant production of the experimental farm School of Veterinary Medicine, Ceará State University, located in Guaiúba, Ceará, in the equatorial zone (4°2ʹ23ʺ S and 38°38ʹ14ʺ W), Brazil. All procedures used in this study were reviewed and approved by the Ethics Committee in Animal Experimentation of Ceará State University (500620/2020).

### Experimental animals, management and treatments

The animals used in this experiment were 21 pluriparous Anglo‐Nubian crossbred adult goats; age and body weight (mean ± SD) were 41.8 ± 6.5 months and 35.5 ± 3.6 kg, respectively. Goats were homogeneously distributed within treatments (P > 0.05) according to their BCS by a trained examiner and assigned a score on a scale of 1–5 with steps of 0.25. BCS was 2.6 ± 0.1 (mean ± SD), equivalent to the subcutaneous fat sternal thickness and kidney fat thickness of 9.7 ± 1.6 mm and 2.0 ± 0.3 mm, respectively. These two anatomical areas were used because of their close relationship with the body condition of goats (Morand-Fehr, 1981; Härter et al., 2014).

**Figure 1:**
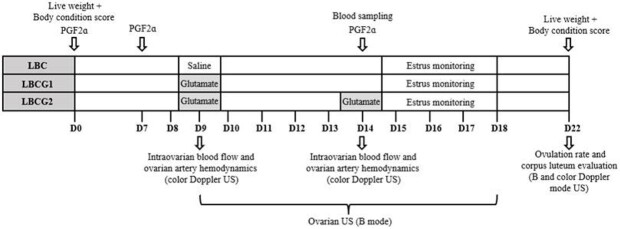
General experimental design including the hormonal protocol and intravenous treatments of goats with low body condition in the control group that received a single dose of saline solution (LBC), and in the groups that received a single dose (LBCG1) or two doses (LBCG2) of glutamate.

To assess the effect of treatments on follicular growth and ovulation, all goats had synchronized estrus and follicular waves as described by Viñoles et al. (2010), by administering three injections of 100 μg of the prostaglandin analog (PGF2ɑ analog) d-cloprostenol (Prolise® - Tecnopec, São Paulo, Brazil) at seven-day intervals (Figure 1). The third application of the PGF2ɑ analog was administered to promote estrus and ovulation simultaneously in all goats.

All animals received the same diet based on Bermuda grass hay and concentrate (ground corn grain, 24%; wheat bran, 12.8%; soybean meal, 1.2%; and mineral and vitamin mixture, 2.0%, based on dry matter of diet). The diet had a roughage: concentrate ratio of 60:40 and was provided to satisfy the nutritional requirements of adult non-dairy goats (NRC, 2007) for breeding. The experimental animals were maintained in collective stalls with free access to mineral supplements and water. The diets were provided twice daily (07:00 and 15:00).

Fourteen goats received 10 mg/kg LW (iv) of glutamate (L-Glutamic acid, SIGMA-ALDRICH – G1251) in a single dose on the 9^th^ day (group LBCG1, n = 7) or two doses: on the 9^th^ and 14^th^ days (group LBCG2, n = 7) (Figure 1). The Glutamate was provided before feeding as a solution prepared by dissolving 1 g glutamate in 100 mL saline solution (10 mg/mL). The baseline diet was maintained for the control group members (LBC; n = 7), and these goats received a saline solution on the 9^th^ day (Figure 1). Glutamate was administered on the 9^th^ day after the emergence of a new follicle wave after the second PGF2ɑ application and on the 14^th^ day at the third PGF2ɑ at ovulation induction.

### Goat in vivo performance traits

For the in vivo performance evaluation, carcass markers were measured, which are represented by subcutaneous and visceral fat. Adipose mass was verified by ultrasonography on days 0 and 22 of the experimental period (Figure 1) by measuring the thickness of the subcutaneous fat deposits at the third sternebrae Teixeira et al. (2008), and to estimate visceral fat, we measured the thickness of kidney fat behind the 13^th^ rib, using the methodology previously described by Härter et al. (2014). A convex transducer with a frequency of 3.5 MHz (model Z5 Vet; Mindray Bio-Medical Electronics Co., Shenzhen, China) was used for kidney imaging. Images were captured in triplicate and measured using a previously calibrated ImageJ program (ImageJ, National Institutes of Health, Millersville, USA). During evaluation, animals were kept stationary, the areas on the right side of the body were shaved, and gel was used as a coupling agent to improve image quality. All goats were weighed on the same dates.

### Ultrasonography analysis

#### Ovarian follicular dynamics and ovulation rate

Ultrasonography was performed once a day from the 9^th^ day to 18^th^ day (Figure 1). Ovarian images were obtained with B-mode ultrasound equipment using a 5 MHz linear transrectal probe (Mindray DP 2200 VET, Mindray Biomedical Electronics Co., Shenzhen, China), as described by Alves et al. (2019). For the analysis of the growth pattern of the ovarian follicles, the ultrasonographic examinations were recorded as videos and using the imageJ program (ImageJ, National Institutes of Health, Millersville, USA), previously calibrated, the mean diameter (average of two dimensions, vertical and horizontal) of each identified follicle was measured. The follicles present in both ovaries were classified according to their diameter in small (< 3 mm), medium (≥ 3 < 5 mm) and large follicles (≥ 5 mm) (Ginther and Kot, 1994).

An ovarian follicular wave was defined as the emergence of a group of small follicles (< 3 mm) that gave rise to one or more large follicles (≥ 3 mm). The day of wave emergence was considered the day when the largest follicle of that wave had reached 3 mm in diameter. The growth phase was defined as the period during which a large follicle grew from 3 mm to its maximum diameter. The regression phase was the period from maximum follicle diameter to a diameter of 3 mm (Viñoles et al., 2010).

Ovulation rate was determined according to Viñoles et al. (2010), with minor modifications. In brief, the collapse of large follicles (> 5 mm) were observed to evaluate the ovulation, followed by an assessment of the presence and count of luteal tissues at the same site eight days later.

##### Intraovarian blood flow and ovarian artery hemodynamics

Evaluations were performed on the 9^th^ day and on 14^th^ day (Figure 1) using a color Doppler ultrasound scanner equipped with a 7.5 MHz linear transrectal probe (model Z5 Vet; Mindray Bio‐Medical Electronics Co., Shenzhen, China). The settings of the scanner (Doppler sampling frequency (PRF) = 1.0 kHz, depth = 6.5 cm, and color gain = 100%) remained constant for the duration of the study. Intraovarian area Doppler was used to assess intraovarian blood flow in the Doppler images, as described by Oliveira et al. (2014), with minor modifications. Briefly, Doppler-mode videos of both ovaries were recorded for subsequent evaluation. From Doppler videos, were selected images containing the cross-sectional area of the ovary with the strongest color Doppler signal, and using the freehand selections function of the imageJ program (ImageJ, National Institutes of Health, Millersville, USA), the ovary was outlined manually to measure the total ovarian area (TA). Subsequently, the intraovarian area Doppler (AD) representing intraovarian blood flow was measured for each ovary. Finally, the intraovarian area Doppler percentage (AD/TA× 100) was obtained for each day and each goat.

To assess the hemodynamics of the ovarian artery, the left and right ovarian arteries were localized immediately after the intraovarian blood flow ultrasound evaluation. The ovarian artery hemodynamics was determined at the most prominent color spot in the ovarian pedicle within 5 mm of the ovarian base. The ovarian artery was identified as a single vessel winding around the ovarian vein. Thus, at this location, the ovarian artery blood flow waveforms were obtained by activating the pulsed Doppler function and placing a Doppler gate with a diameter of 2 mm over the colored ovarian artery. The values of end-diastolic velocity, peak systolic velocity and hearth rate were obtained automatically by the ultrasound equipment from two spectral Doppler waves. The angle of insonation between the Doppler ultrasound beam and flow direction in the ovarian artery was 60° (Souza et al., 2016).

##### Corpus luteum evaluation

Luteal activity was measured using B-mode ultrasonography and color Doppler on the 22^nd^ (Figure 1). Briefly, B-mode and Doppler-mode videos of both ovaries were recorded for subsequent evaluation. Evaluations were performed using the B-mode ultrasound equipped with a 5.0 MHz linear transrectal probe for identification the corpus luteum (CL). From the B-mode, the frozen images were used for CL count and to calculate luteal volume (m^3^) (calculated using the sphere formula) (Balaro et al., 2017). Color Doppler ultrasound was performed to assess the CL blood perfusion, measuring the luteal Doppler area as described by Balaro et al. (2017), with minor modifications. To obtain the Doppler area, a color Doppler scanner equipped with a 7.5 MHz linear transrectal probe with PRF = 1.0 kHz, depth = 6.5 cm, and color gain = 100% was used. Initial identification of the CL was made with B-mode and then the CL video was made with color-Doppler function. With images corresponding to the cross-section of the CL at its greatest diameter and using the freehand selections function of the previously calibrated ImageJ program (ImageJ, National Institutes of Health, Millersville, USA), the total area of the CL and the luteal Doppler area were measured, then with these values the percentage of the Doppler area was measured.

### Estrus behavior monitoring

Estrus behavior was monitored using a buck, which was introduced four times daily in the pen to detect estrus (06:00 AM, 10:00 AM, 14:00 PM, and 18:00 PM h) for three days, beginning 12 hours after the third administration of PGF2α (Figure 1). The onset of estrus was considered when the goats showed receptivity to the buck, and the end when showed no more receptivity. These data were then used to determine the estrus length.

### Metabolites and Glutathione Peroxidase (GPx) assays

Blood samples were collected on the 14^th^ day, using heparinized vacutainer tubes (Labor import, Wei Hai, China) before morning feeding. The samples were centrifuged at 600 × *g* for 15 min, and the plasma obtained was stored at −20 °C for further quantification of metabolites. Plasma concentrations of glucose and cholesterol were determined using an automated biochemical analyzer (Mindray BS 120, Mindray®) and commercial kits (Bioclin, Quibasa, Minas Gerais, Brazil). The sensitivity of the assay kit was 1.5088 mg/dL for glucose and 1.472 mg/dL for cholesterol. GPx was analyzed using a semi-automatic biochemical analyzer (Randox RX Monza TM, Randox Laboratories, Crumlin, UK) and commercial kits (Randox Laboratories, Crumlin, UK) with a sensitivity of 75 U/L.

### Statistical analysis

Statistical analyses were performed using Statistica Software, version v. 13.4.0.14 (2018; TIBCO Software, Inc., Palo Alto, CA, USA). Data were initially verified for normality and homoscedasticity assumptions by Kolmogorov–Smirnov and Bartlett’s tests respectively. When homoscedasticity condition was not respected, data were transformed to the log10x.

Data on in vivo performance and dynamic follicles were subjected to analysis of variance (ANOVA) using the GLM procedures in a factorial arrangement, where the main effects tested were the group (LBC, LBCG1, or LBCG2), the effect of the interval of assessment used (time), and interaction (group vs. time). Data on estrus response, metabolite levels, ovulatory rate, and corpus luteum response were analyzed using GLM procedures. The “group” was included as a factor for the model. Descriptive ultrasonography data regarding carcass markers, intraovarian blood flow, and ovarian artery hemodynamics were obtained using the GLM procedures for repeated measures of ANOVA. The effects tested were the group, the effect of the interval of assessment used (time), and interaction (group vs. time). The recorded anatomical images (1, 2, and 3) were repeated. All pairwise comparisons were performed using the Newman–Keuls post hoc test, which was applied when ANOVA indicated a significant difference (P < 0.05).

The number of goats in the estrous effect group was analyzed using the Kruskal–Wallis ANOVA test, and comparisons were performed using the Chi-square test, with P *<* 0.05 indicating statistical significance.

## Results

### In vivo performance

No significant effect of group on the live weight of the animals or carcass markers was observed. For the in vivo performance traits, no significant interaction between the evaluation interval and the groups was detected (P > 0.05) (Table 1).

**Table 1:** Means and standard errors of body weight, subcutaneous sternal fat thickness, kidney fat thickness, metabolites and estrus response to hormonal protocol in goats with low body condition score treated with one or two fixed doses of glutamate.

**Attributes**	**Groups**				**p Value**		
	**LBC**	**LBCG1**	**LBCG2**	**SEM**	**Group**	**Time**	**G vs. T**
*Body weight and carcass markers*							
Body weight, kg	36.6	34.2	35.9	0.292	0.0631	0.2887	0.9205
SSFT, mm	10.1	10.3	10.1	0.157	0.7437	0.2243	0.9371
KFT, mm	2.1	2.2	2.0	0.037	0.1231	0.4626	0.3118
*Metabolites* ^*^							
Glucose, mg/dL	55.7	51.7	53.2	0.733	0.1331	-	-
Cholesterol, mg/dL	61.6	65.0	64.1	1.185	0.6535	-	-
Glutathione peroxidase, U/L	325.7	326.2	327.0	1.018	0.3315	-	-
*Estrus response*							
No of does in estrus, % (n\n)	100 (7\7)	86 (6\7)	57 (4\7)	-	0.7843	-	-
Onset estrus, h	36.3	35.2	45.2	3.524	0.9034	-	-
Estrus length, h	56.7	52.7	70.0	3.403	0.1411	-	-
Ovarian artery hemodynamics*							
Peak systolic velocity, cm/s	35.6a	37.3a	53.7b	2.198	0.0144	-	-
End-diastolic velocity, cm/s	9.5a	12.2a	16.4b	1.281	0.0384	-	-
Hearth rate, bpm	65.0a	71.4a	80.5b	2.037	0.0085	-	-

Abbreviations: SEM: standard error of mean; SSCFT: subcutaneous sternal fat thickness; KFT: kidney fat thickness. *Performed at 14^th^ Day. Time, ANOVA effect for interval of assessment used.

### Glucose, cholesterol, and GPx levels

The plasma concentrations of cholesterol, glucose, and GPx measured at ovulation induction on the 14^th^ day are shown in Table 1. No differences were observed among the means of the groups.

### Estrus synchronization and ovarian response

**Figure 2:**
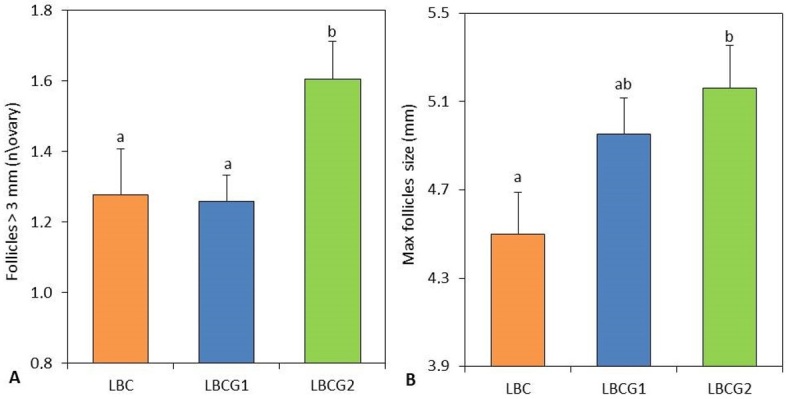
Number of follicles > 3 mm (left **figure A)** and maximum follicles size (right **figure B),** determined by ultrasonography during the experimental period in goats with low body condition score untreated (LBC) and treated with one (LBCG1) or two (LBCG2) fixed doses of glutamate. Values are expressed as mean ± standard error of the mean. ^a,b^ P < 0.05 differences between groups.

**Figure 3:**
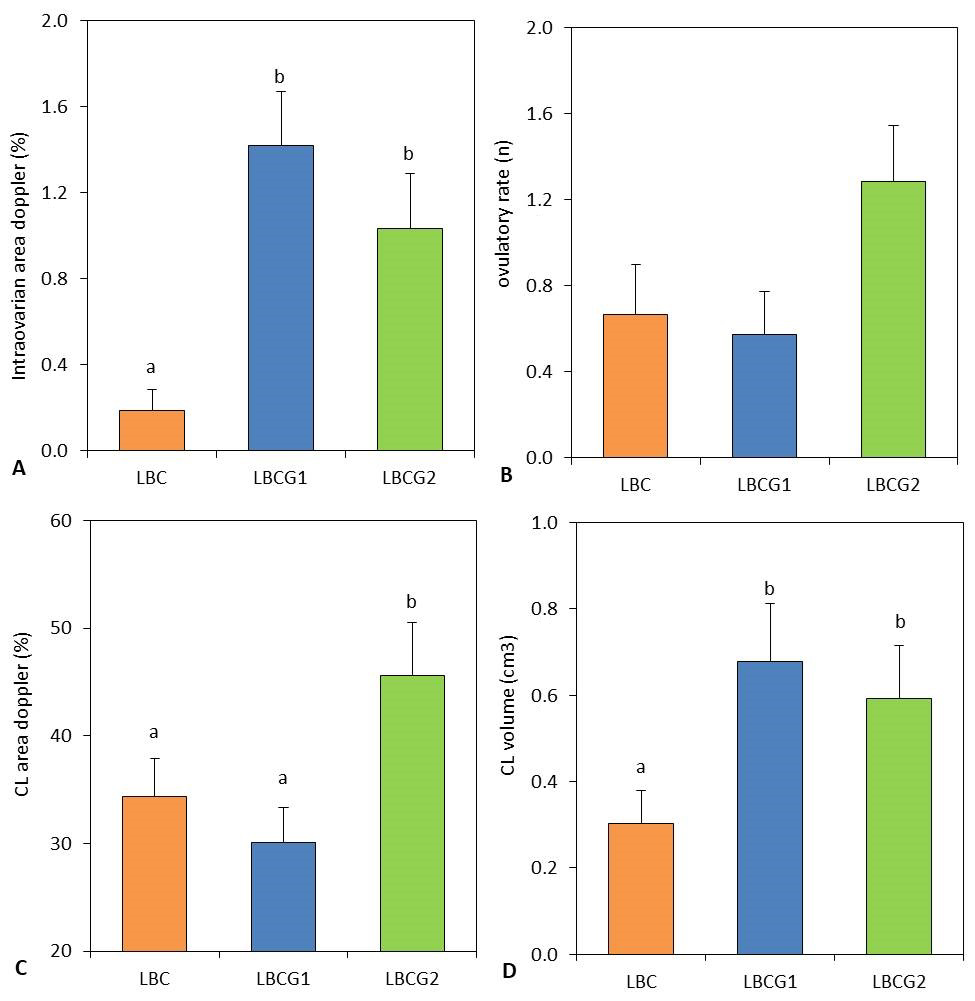
Ovarian color Doppler intraovarian area measured on 9^th^ day and 14^th^ day (Upper **figure A),** ovulation rate (Upper **figure B),** Doppler color area of corpus luteum (Bottom **figure C)** and estimated volume of corpus luteum (Bottom **figure D),** measured on 22^th^, in goats with low body condition score untreated (LBC) and treated with one (LBCG1) or two (LBCG2) fixed doses of glutamate. Values are expressed as mean ± standard error of the mean. ^a,b^ P < 0.05 differences between groups.

No hormonally induced estrus response parameters showed significant differences between the experimental groups (Table 1). The LBCG2 group registered a higher (P < 0.05) number of follicles with a diameter > 3 mm than the other groups, and a significant increase (P < 0.05) in the maximum follicular diameter was observed in this group compared with that in the saline group (Figure 2). The LBCG2 group also showed an ovulation rate twice that of the other treatment groups (1.3 vs. 0.6); however, no statistical significance was found in the comparison between means (P > 0.05) (Figure 3B).

### Intraovarian area Doppler and hemodynamics values

Figure 3A shows the Doppler area of intraovarian blood flow measured on the day of glutamate administration. In the LBCG1 and LBCG2 groups, a significant increase (P < 0.05) of intraovarian blood flow was observed. On the day of the second glutamate administration (day 14), a higher systolic and diastolic ovarian artery peak velocity (P < 0.05) and a higher heart rate (P = 0.0085) were also observed in the LBCG2 group (Table 1).

### Luteal size and luteal Doppler vascularized area

Figure 3 illustrate the values related to the estimated volume of the corpus luteum and their Doppler area of blood perfusion. Luteal volume was higher in the two groups supplemented with glutamate (P < 0.05) than in the saline control, and the percentage of the Doppler area was greater in the LBCG2 group than the in other two groups (P < 0.05).

## Discussion

Neuronal activity controls endocrinological and hormonal changes during the estrous cycle via the intervention of several AAs that act as neurotransmitters (Cao et al., 2021). Glutamate is the main neurotransmitter that promotes neuroendocrine activity during reproduction in mammals (Chiang and Park, 2020). GnRH release is stimulated by glutamate, and glutamate activity is regulated by P4 and E2 depending on the phase of the estrous cycle promoting FSH release or LH pulses (Barth et al., 2015; Porter et al., 2021).

Our results showed that glutamate represents an alternative nutritional supplementation for ovarian stimulation in animals with a low body condition score.

Repeated administration of glutamate at the proposed dosage was more efficient in terms of the number of large follicles (> 3 mm), vascularization, follicular size, and the quality of the corpus luteum than a single application. Additionally, the reproductive response of the groups supplemented with AA occurred without apparent changes in the concentrations of peripheral circulating metabolites and without variations in oxidative stress, body weight, or adipose deposition.

Glutamate is an AA that acts as an excitatory neurotransmitter and stimulates GnRH production and release (Chiang and Park, 2020). Under normal conditions, its activity is regulated by steroid hormones (Barth et al., 2015; Porter et al., 2021), and supplementation with this amino acid has been shown to be effective in reducing the negative feedback effects of E2 (Luna-García et al., 2022).

The role of glutamate has been reported in the protocol for intravenous administration of 0.175 mg/kg LW of L-glutamine in cyclic goats improved follicular development and ovulation rate, because glutamate is particularly responsible for the production of the preovulatory peak of GnRH, by the activity of factors such as Kisspeptin, estradiol and the expression of glutamate receptors in the brain (Meza-Herrera et al., 2014b), and during seasonal anestrus, 12 doses of 7 mg/kg of L-glutamine were administered. LW acted on the factors that promoted negative feedback, reducing its effects on the HPO axis, proving that glutamate intervenes as a signal for the beginning of the breeding season (Meza-Herrera et al., 2020). As described by Luna-García et al. (2022), glutamate possibly acts by decreasing the action of dopamine on kisspeptin-producing neurons, activating the hypothalamic centers that synthesize and release GnRH, which promotes an increase in LH pulses and acts on ovarian activity.

Metabolic signals observed in animals with adequate body weight and BCS are not known to influence the effects exerted by glutamate because interactions between these factors have not been reported (Torres-Moreno et al., 2009; Meza-Herrera et al., 2020; Luna-García et al., 2022). We observed that the action of glutamate was not accompanied by changes in glycemia or cholesterol in animals with low BCS, suggesting that the involvement of other metabolic signals in addition to those of live weight and BCS may be related to ovarian reactivation in goats supplemented with glutamate (Meza-Herrera et al., 2020).

The main objective of this study was to verify whether glutamate supplementation in animals with low nutritional status improved ovarian response. To achieve this goal, we used a higher dosage than that prescribed in the literature, and proposed a simpler protocol than existing ones to reduce the number of applications and time interval between AA administrations.

The observed results were related to high intraovarian vascularization in both supplemented groups and to the increase in the hemodynamic values of the ovarian artery during ovulation induction in LBCG2 group members.

Follicular activity depends on angiogenesis and ischemia, factors that determine the physiological state (Jiang et al., 2003; Kordowitzki et al., 2021). Angiogenesis at the theca level gives rise to a microcapillary network of blood vessels that supplies the follicle with the substances and oxygen necessary to promote its growth and steroidogenic function (Jiang et al., 2003; Pancarci et al., 2012; Viana et al., 2013). Thus, in both glutamate-treated groups, there was a significant increase in the maximum follicular diameter.

Administration of l-glutamine stimulated an increase in LH pulses (Luna-García et al., 2022). LH promotes androgen biosynthesis in theca cells during E2 synthesis in granulosa cells (Hattori et al., 2018). E2 production occurs during the follicular growth, selection, and dominance phases of follicular dynamics, where angiogenic activity is most intense (Acosta et al., 2003; Matsui and Miyamoto, 2009; Arashiro et al., 2018); the metabolic activity of the follicles also increases, requiring an increase in blood supply, which increases the hemodynamic values of the ovarian artery (Witt et al., 2012; Oliveira et al., 2019). LH stimulates androgen production in theca cells and increases FSH sensitivity in the granulosa cells of small antral follicles, also facilitates follicular development and survival by activating the IGF system which suppresses granulosa cell apoptosis and follicular atresia, allowing the development and maturation of more follicles (Hattori et al., 2018). In our study, we observed that the goats in the supplemented groups developed follicles with a larger diameter than those in the control group, and the goats that received a double dose of glutamate developed a greater number of follicles with a diameter > 3 mm than those in the control group.

Glutamate supply also presents advantages in relation to luteal quality. Both supplemented groups showed a high volume of the CL, and, in parallel, the Doppler ultrasonographic evaluation indicated high luteal blood perfusion in the animals that received a double dose of glutamate.

Follicles with large diameters are known to produce more estrogen, intensify the behavioral symptoms of estrus, produce larger CLs with the ability to produce higher concentrations of P4 (Hameed et al., 2020; Oosthuizen et al., 2020), and are characterized by higher vascular activity (López-Gatius and Garcia-Ispierto, 2022). These changes may have occurred in the supplemented groups because of the effect of glutamate on follicular development. In CL, there was a strong association between vascularization and steroidogenic functionality, because the growth of luteal tissue and P4 production depend on the development of blood vessels that carry the necessary blood and oxygen (Balaro et al., 2017; López-Gatius and Garcia-Ispierto, 2022). Balaro et al. (2017) classified functional CL as those that are able to maintain P4 concentrations above 1.0 ng/mL and that present at least 30% of the area of colored pixels in goats evaluated with Doppler ultrasonography.

The low quality of the CL is one of the main factors responsible for the reduction in pregnancy rates (Hameed et al., 2020; Oosthuizen et al., 2020). Various environmental and individual factors can cause luteal insufficiency as a result of CL originating from a low-quality follicle, affecting the outcomes of assisted reproductive biotechnologies (Al Yacoub et al., 2011; Lima et al., 2020; Nanas et al., 2021). Suitable luteal functionality prepares the endometrium for the development of pregnancy before embryo implantation, acting as a mediator in the differentiation and secretion of endometrial glands (Lonergan, 2011; Patel et al., 2015) and in endometrial angiogenesis (Bazer et al., 2009; Patel et al., 2015). These events allow the embryo to elongate and subsequently produce interferon-tau, which allows the maternal recognition of pregnancy in ruminants (Mann and Lamming, 2001).

## Conclusion

Administration of 10 mg/kg PV glutamate in two doses every five days showed to be an effective treatment for ovarian stimulation in goats with low body condition scores.

## Data Availability

All data generated and analyzed during this study are included in this article.
